# Short-term prognosis analysis of critically ill patients entering the emergency department by different ways based on propensity score matching

**DOI:** 10.1097/MD.0000000000045502

**Published:** 2025-11-28

**Authors:** Xia Ji, Ying An, Ya-Mei Guan, Na Liu, Dan-Dan Zhu, Yan-Hua Ma

**Affiliations:** a Emergency Department, Beijing Tongren Hospital, Capital Medical University, Beijing, China; b Nursing Department, Beijing Tongren Hospital, Capital Medical University, Beijing, China; c Department of Dermatology Venereology, Beijing Tongren Hospital, Capital Medical University, Beijing, China.

**Keywords:** ambulance, critically ill patients, different transportation methods, propensity score matching, self-admission, short-term prognosis

## Abstract

The aim was to explore the short-term prognosis of critically ill patients admitted to the emergency department (ED) by 2 different means of transportation (ambulance and nonambulance), aiming to identify critically ill patients and ensure patient safety. Critically ill patients admitted to the ED from January 1, 2020, to December 31, 2020, were included and divided into 2 groups: those with ambulance transportation and those with nonambulance transportation. The 2 groups were matched via the propensity score matching method, and the short-term prognoses of the 2 groups when the patients left the ED were compared. A total of 1150 patients were enrolled, including 713 (62.0%) male and 437 (38.0%) female patients with a median age of 70 (58, 83) years. Patients aged 70 years and older who were transported by ambulance had longer stays (*P* = .023), experienced a greater number of resuscitations within 24 hours (*P* = .007) and were more likely to experience in-hospital death or intensive care unit admission (*P* = .007) than those with nonambulance transportation. Compared with patients with nonambulance transportation, patients with ambulance transportation who were admitted to the ED or who died within 24 hours after discharge from the hospital had more rescue visits and longer stays in the emergency room, and a greater number of these patients were transferred to the ward unit or the hospital. More attention should be given to the management of patients with ambulance transportation to reduce the risk of adverse nursing outcomes.

## 1. Introduction

The emergency department (ED) serves as a critical access point in hospitals for the daily diagnosis and resuscitation of a wide array of critically ill patients. These patients, which span all age groups, are frequently admitted with various traumatic, acute cardiovascular, and cerebrovascular emergencies characterized by rapid onset, rapid progression, high mortality, and the potential for immediate life-threatening conditions. The admission method for a patient is determined by their physiological state at the time of the event and their on-scene condition. In this study, patients were categorized into ambulance and nonambulance transportation groups. Patients with sudden and severe symptoms are most often admitted through ambulance services. International research indicates that using emergency medical services (EMSs) significantly reduces prehospital delays, providing quicker treatment for conditions such as stroke.^[1,2]^ A national study in China in 2018 revealed that the average usage rate of EMSs among acute stroke patients was 12.5%.^[3]^ A survey in the United States revealed that 60% of patients with acute ST-elevation myocardial infarction (STEMI) utilized EMSs, which ensured immediate access to a 12-lead ECG and reduced the time to reperfusion.^[4]^ Patients who walked to the hospital and were admitted are likely to have milder conditions. Upon admission, medical personnel triage patients on the basis of established criteria,^[5–7]^ directing them to appropriate care settings. In China, many EDs adopt a four-level preexamination triage standard. Triage personnel classify patients as follow: level I (critical) or level II (severe) patients, who are treated in the resuscitation room; level III (urgent) patients, who are directed to the examination room for immediate care; and level IV (nonurgent) patients, who have less severe, common conditions and wait for treatment.

From a clinical practice perspective, there are discernible differences in the short-term outcomes of critically ill patients between the 2 transportation methods, including variations in the length of stay in the resuscitation room, the frequency of resuscitation within the first 24 hours of admission, the likelihood of being transferred to the intensive care unit (ICU), and the likelihood of death.^[8]^ Therefore, it is necessary to further analyze the short-term prognosis of these 2 patient groups to identify differences and similarities in the prompt implementation of accurate assessments and corresponding interventions, thus effectively improving patient prognosis. These findings have significant implications for the recovery of critically ill patients, yet research on this topic is scarce.

Various assessment tools, such as the rapid emergency medicine score,^[9]^ National early warning score,^[10]^ Acute Physiology and Chronic Health Evaluation (APACHE II),^[11]^ and the modified early warning score,^[12]^ are utilized to evaluate the prognosis of patients in the ED. Researchers have also identified independent risk factors affecting the prognosis of critically ill patients in the ED, developing rapid prognostic scoring tools and predictive models for their outcomes.^[13]^ Some researchers have tested various interventions to improve the outcomes of critically ill patients in the ED, such as leveraging the medical resources of surrounding primary hospitals through ambulance transport based on chest pain centers^[14]^ and establishing cross-hospital rapid response mechanisms to increase patient treatment efficiency, forming a regional collaborative care system.^[15]^

Reports on the prognosis of critically ill patients admitted to the ED through different transportation methods are limited. In most cases, ambulances can significantly improve patient outcomes rather than merely reflecting the severity of the disease. For example, patients with acute ischemic stroke who are transported by an ambulance have a shorter time from onset to thrombolysis, reduced hospitalization delays, improved reperfusion rates, and better outcomes^[16]^; among STEMI patients, those transported by an ambulance have higher rates of reperfusion therapy and lower rates of adverse cardiovascular events.^[17]^ These findings indicate that ambulance transport itself is not an independent risk factor for a poor prognosis but rather a critical component in improving outcomes by shortening the treatment time and optimizing prehospital interventions. Therefore, this study aimed to group and compare the outcome indicators of patients brought to our ED by ambulance and by other means. Owing to various confounding factors that may bias the results, affecting their credibility, we employed propensity score matching (PSM). This method synthesizes the effects of multiple covariates into a single propensity score, which is used to balance the covariate distribution between intervention and control groups, thereby controlling for confounding biases.^[18]^

Reasonable PSM between groups reduces the interference of confounding factors, enhancing statistical power and reducing data errors.^[19]^ For this reason, we used PSM to clarify the short-term outcomes of critically ill emergency patients admitted to the ED through different transportation methods, which will provide a basis for the early treatment of such patients.

## 2. Background/literature

This study was conducted in a large comprehensive hospital in Beijing, China. This hospital was established in 1886 and is recognized for its national key disciplines in ophthalmology and otolaryngology. The hospital has over 3600 employees, 62 clinical and medical technical departments, 1611 operational beds, and an annual emergency and outpatient volume exceeding 2.7 million patients. The hospital has 2 campuses in Beijing, located in the city center and the economic and technological development zone, each with an ED. This research was carried out at the ED of the central city campus.

## 3. Methods

### 3.1. Setting and Sample

By convenience sampling, we gathered data from critically ill patients admitted to our ED’s resuscitation room between January 1, 2020, and December 31, 2020. The inclusion criteria were as follows: aged over 14 years; admitted to the resuscitation room; and initially triaged as level I (critical) or level II (urgent) during emergency prescreening triage, including level I to II reclassifications during the visit. The exclusion criterion was incomplete data records.

### 3.2. Instrument

The data collection form, created by our team, included sections on patient demographics, vital signs, and short-term outcomes.

#### 3.2.1. Patient demographics

This section covered sex, age, department in charge, method of transportation, admission time, and length of stay in the resuscitation room. The length of stay in the resuscitation room referred to the time from when a patient entered the emergency resuscitation room to the time when they left. This time was calculated in hours, with any period ≥30 minutes rounded to 1 hour and periods <30 minutes excluded from this study.

#### 3.2.2. Vital signs

Respiration, pulse, systolic blood pressure, temperature, and peripheral oxygen saturation measurements were taken upon the patient’s arrival to the resuscitation room. All vital sign data were monitored and recorded via a surveillance device.

#### 3.2.3. Short-term outcomes

This referred to a patient’s outcome upon leaving the resuscitation room, categorized as stable (remaining in the resuscitation room, hospitalization, surgery) or critical (transfer to the ICU or death).

### 3.3. Procedure

#### 3.3.1. Phase one

Retrospective data were collected for critically ill patients admitted to the ED through different transportation methods from January 1, 2020, to December 31, 2020, who met the inclusion and exclusion criteria.

#### 3.3.2. Phase two

The data were collected and entered into a specially designed form.

#### 3.3.3. Phase three

PSM and statistical analysis were conducted.

### 3.4. Statistical analysis

IBM SPSS 25.0 was used for PSM and statistical analysis. Logistic regression was employed to calculate the propensity scores, and nearest neighbor matching was applied at a 1:1 ratio, with the method of transportation as the group indicator. The predictors included sex, age, shift of admission, and department in charge, with a matching tolerance of 0.02. At the same time, a sensitivity analysis was carried out by adjusting the matching tolerance to 0.01. Nonnormally distributed measurement data are described as medians and quartiles and were compared via the Mann–Whitney *U* test; categorical data are described as frequencies and percentages and were compared via the χ^2^ test, with *P* < .05 denoting statistical significance.

### 3.5. Ethical considerations

The research protocol was approved by the hospital’s ethics committee (code: ***). Identifiable information (e.g., names, ID numbers) was not used in the analysis or discussion, as it was strictly confidential and solely used for scientific research. All relevant ethical standards for research were met.

## 4. Data/results

### 4.1. Patient characteristics

When the matching tolerance was set to 0.02, this study included a total of 1150 patients, of whom 713 (62.0%) were males and 437 (38.0%) were females. The median age of the patients was 70 years, and the ages ranged between 58 and 83 years. There were 365 patients transported by ambulance and 785 transported by other means. Before PSM, significant differences in age and the department in charge were found between the 2 groups. PSM yielded 362 pairs of patients. After matching, all the baseline data were comparable (Table 1) and were included in the statistical analysis. When the matching tolerance was set to 0.01, a total of 723 patients were included, of whom 365 were brought by ambulances and 358 were brought by nonambulance transportation.

**Table 1:** Comparison of patient data before and after matching.

Item	Before matching (N = 1150)					After matching (N = 724)			
	Non-ambulance admission (n = 785)		Ambulance admission (n = 365)	Statistics	*P* value	Ambulance admission (n = 362)	Ambulance admission (n = 362)	Statistics	*P* value
Age [yr, M (P25, P75)]	69 (57, 82)		74 (61, 86)	−4.294	<.001	74 (63, 84)	74 (61, 86)	−0.347	.728
Sex (% cases)									
Male	481 (61.3)		232 (63.6)	0.553	.457	230 (63.5)	230 (63.7)	0.218	.641
Female	304 (38.7)		133 (36.4)			132 (34.6)	132 (36.5)		
Admission shift (% cases)									
Day shift	312 (39.7)		134 (36.7)	0.999	.607	138 (38. 1)	133 (36.7)	0.180	.914
Evening shift	403 (51.3)		198 (54.2)			194 (8.3)	197 (54.4)		
Night shift	70 (8.9)		33 (9.0)			30 (8.3)	32 (8.8)		
Managing departments									
Internal medicine department	627 (79.9)		266 (72.9)	7.027	.008	266 (73.5)	266 (73.5)	0.000	>.99
Non-internal medicine first vital signs in resuscitation room	158 (20. 1)		99 (27. 1)			96 (26.5)	96 (26.5)		
Temperature [°C, M (P25, P75)]	36.5 (36.3, 36.7)		36.5 (36.3, 36. 7)	−0.192	.848	36.5 (36.2, 36.8)	36.5 (36.3, 36.7)	−0.072	.943
Pulse [beats/min, M (P25, P75)]	90 (74, 100)		89 (74, 107)	−0.736	.462	91 (75, 110)	89 (74, 107)	−1.023	.306
Respiration [beats/min, M (P25, P75)]	20 (19, 23)		21 (18.25, 25)	−1.942	.052	20 (19.24)	21 (18, 25)	−1.273	.203
Systolic pressure [mm Hg, M (P25, P75)]	130 (110, 151)	134 (112, 158)		−1.672	.095	134 (112.75, 155.25)	134 (111.75, 158)	−0.564	.573
Blood oxygen [%, M (P25, P75)]	100 (97, 100)	99 (96, 100)		−1.354	.175	99 (96, 100)	99 (96, 100)	−0.625	.532

### 4.2. Comparison of the main outcomes between groups after PSM

When the matching tolerance was set to 0.02, 2 sets of data were obtained. After matching, the patients transported by ambulance stayed in the resuscitation room longer (*P* = .023), experienced a greater number of resuscitations within the first 24 hours of admission (*P* = .007), and were more likely to have experienced in-hospital death or transfer to the ICU (*P* = .007) (Table 2).

**Table 2:** Comparison of the main outcomes between groups after matching (N = 724).

Item	Ambulance admission (n = 362)	Ambulance admission (n = 362)	Statistics	*P* value
Resuscitation room stay duration	14 (5, 44.5)	20.5 (6, 49)	−2.272	.023
Condition status				
Stable group	272 (75. 1)	239 (66.0)	7.244	.007
Critical group	90 (24.9)	123 (34.0)		
Resuscitation within 24 h of admission				
Yes	81 (22.4)	195 (29.0)	4.167	.041
No	281 (77.6)	257 (71.0)		

When the matching tolerance was set to 0.01, 2 sets of data were obtained as follows: the patients admitted by an ambulance stayed in the emergency room for 21 (6, 49.5) hours, whereas those admitted without an ambulance stayed for 11 (4, 37.25) hours (*Z* = −3.614, *P* < .01). The former group included 123 patients (33.7%) with more severe conditions than those in the latter group (56 patients, 15.6%) (χ^2^ value = 31.631, *P* < .01). Additionally, 105 patients (28.8%) in the former group required resuscitation within 24 hours, which was more than the 48 patients (13.4%) in the latter group (χ^2^ value = 25.556, *P* < .01). Therefore, in the sensitivity analysis, there was still a statistically significant difference in the primary outcomes between the 2 groups of patients.

## 5. Discussion

### 5.1. Longer resuscitation room stays in patients transported by ambulance

After adjusting for confounders by PSM, the present study indicated that patients transported by ambulance had a significantly longer median stay in the resuscitation room (20.5 hours) than those admitted not transported by ambulance (14 hours). Possible reasons include the following: Patients who are transported by ambulance often have sudden disease onset, rapid disease progression, and severe conditions. This study revealed that ambulance transport could expedite treatment for acute stroke patients and reduce prehospital delays; however, it did not shorten the duration of in-hospital treatment, which is consistent with the findings of Kim et al^[20]^; Many critically ill patients transported by ambulance present with complex conditions, some with hidden symptoms, making immediate diagnosis challenging. These patients often need to stay in the resuscitation room for a long time to undergo comprehensive diagnostics and management. Delays in testing can prolong hospital stays.^[21]^ These patients often have unstable initial conditions that pose significant risks during transfer^[22]^ and necessitate continuous monitoring and treatment in the resuscitation room until stabilization, which inevitably extends their stay; and Several studies^[23,24]^ have indicated that patients who are transported by ambulance often present with conditions involving multiple systems and require the collaboration of various departments beyond the ED for treatment.

It is often challenging for a specific specialty to admit these patients immediately, leading to prolonged stays in the resuscitation room while awaiting more specialized inpatient care. Currently, hospitals in the country all adopt the grading standards that were set by the National Health and Family Planning Commission.^[25]^ The triage system relies primarily on patients’ vital signs as an objective basis. The existing regulations do not specify detailed criteria for different levels of critical cases transported by ambulances, which may lead to resource congestion. Critically ill patients tend to prefer ambulances,^[26]^ with their clinical presentation being relatively high risk at admission. Patients transported by ambulances are often assumed to have priority, but there can be delays in practice. Ambulances must immediately attend to patients upon arrival at the ED, but if multiple critically ill patients (such as those in mass casualty incidents) arrive simultaneously, resource allocation may face pressure. Additionally, ambulances being involved in nonemergency needs can delay emergency responses,^[27]^ indirectly increasing the risks for critically ill patients. Therefore, it is necessary to refine the classification of ambulance priorities, further categorize cases into “immediate life threat,” such as cardiac arrest, and “potential high risk,” such as chest pain with abnormal ECG results, and develop corresponding resource allocation strategies. First, training for emergency personnel should be strengthened to enhance their medical skills and emergency response capabilities, ensuring that they can provide more precise care during transportation and buying time for subsequent treatment, thereby improving survival rates.

Second, the ambulance dispatch systems should be optimized to shorten response times and ensure that ambulances can reach patients quickly. Ambulances notify patients of preliminary information in advance during transportation, allowing hospitals to understand the patient’s condition early and make adequate preparations, achieving seamless integration between prehospital emergency care and in-hospital treatment. This enables hospitals to activate relevant departments for green channel preparation in advance. Establishing a regional collaborative rescue model is key to improving the success rate of acute STEMI treatment.^[28,29]^ EMS transportation combined with the green channel model can reduce handover delays and waiting times to some extent, further alleviating congestion in the emergency room. However, there may be response delays in actual operations, and the connection between the EMS and in-hospital green channel may not be tight enough, leading to some patients still facing delays upon arrival at the hospital.^[30]^ Hospitals need to improve systems and mechanisms, allocate emergency resources reasonably, and require all departments to prioritize cooperation with emergency rescue efforts. An emergency priority system should be established to ensure that critically ill patients can be rapidly transferred to the ICU or operating room. This not only improves treatment efficiency but also reduces the time that patients spend in the emergency room, effectively easing congestion. Additionally, under policy support, investment in ambulance equipment should be increased. Ambulances equipped with advanced devices can provide better life support for patients, further enhancing overall emergency outcomes. These measures are expected to significantly improve the prognosis of patients transported by ambulances. This also reminds clinical nursing staff to pay more attention to patients being transported by an ambulance, further improving the efficiency of such patient visits and emergency management capabilities.

This complexity in patient care necessitates a highly coordinated approach among different medical specialties to optimize treatment pathways and minimize the time spent in the emergency resuscitation area.

### 5.2. More frequent resuscitation within 24 hours for patients transported by ambulance

Within 24 hours of admission, 81 patients with nonambulance transportation required resuscitation, compared with 195 patients transported by ambulance, indicating a greater incidence of resuscitation among the latter group. This might be related to the fact that patients who are transported by ambulance are typically in worse condition and more critically ill than those who arrive at the hospital by other means. Despite initial treatment, these patients remain unstable and are at high risk of sudden health deterioration, necessitating resuscitation within 24 hours.

The possible reasons include the following: Among patients who require resuscitation within 24 hours, patients with cardiovascular and cerebrovascular diseases are critically ill and are mostly transported by ambulance, often being directly admitted for resuscitation care as level I or II patients. These patients usually need immediate life-saving interventions due to their prehospital critical conditions, such as cardiac arrest,^[31,32]^ which requires urgent CPR and other emergency measures upon arrival; Patients with severe conditions, such as stroke, are more likely to be transported by ambulance. Multiple studies from developed countries have demonstrated that the use of EMSs significantly reduces prehospital delays and facilitates faster treatment for stroke patients.^[1,2]^ Upon ED admission, these patients typically undergo urgent thrombolytic therapy, and those with severe conditions require the immediate implementation of various resuscitation measures. Therefore, it is essential for emergency medical teams, including ambulance teams, emergency receivers, and resuscitation room nurses, to coordinate care effectively, ensuring continuous monitoring and readiness to respond to rapid changes in a patient’s condition to increase the likelihood of recovery.

### 5.3. Higher probability of condition instability in patients transported by ambulance

This study revealed that patients transported by ambulance, compared with those who self-presented to the hospital, experienced more rapid progression of their medical conditions, leading to a greater likelihood of being transferred to the ICU for further treatment or experiencing in-hospital death. The in-hospital mortality rate for patients transported by ambulance is greater than that for patients with other modes of transportation, particularly for critical patients with conditions such as cardiovascular diseases,^[33]^ traumatic brain injuries,^[34]^ acute poisoning,^[35]^ and acute exacerbations of chronic diseases, especially in the elderly population.^[36,37]^ These patients often have unstable vital signs, and their outcomes are influenced by various factors, including the timing of arrival at the ED, the severity of the injury, and the patient’s physiological state, making ICU admission and death unlikely outcomes of emergency care. This highlights the importance of careful observation and assessment of patients transported by ambulance by nursing staff and the need for proactive medical and specialized nursing interventions. Such actions can significantly impact the ability of medical teams to provide timely and appropriate treatment and ultimately improve the care outcomes and quality of life of patients.

### 5.4. Limitations of the study

This study has certain limitations. First, the study was conducted at a hospital in Beijing, and its results are limited in generalizability because of differences in the accuracy and efficiency of triage systems across different regions, which may affect the priority and outcomes of patients admitted through these 2 different admission methods. Additionally, variations in hospital capacities can impact patient waiting times and treatment outcomes, all of which could influence the short-term prognosis. Therefore, future research should consider further validation and exploration at different medical institutions and in different regions. Second, the data used in this study compared ambulance and nonambulance hospital admissions without analyzing how self-transportation modes might affect patient outcomes. The changes in outcomes for critically ill patients who were transported within subcategories of the nonambulance group (such as private vehicles, taxis, or public transportation) were not examined in this study. Therefore, future analyses could consider the impacts of different self-transportation modes on risk factors for critically ill patients.

## 6. Conclusions

In conclusion, an analysis of the relationships between the means of hospital arrival and the severity of patients’ conditions revealed that ambulance use was often associated with increased severity, mortality rates, and ICU admission rates. Additionally, it is necessary to enhance prehospital assessment, optimize prehospital emergency care, expand the use of mobile stroke units, shorten emergency response times, and reduce transport delays. For critically ill patients admitted by ambulances, regional collaborative treatment models should be strengthened to improve the EMS combined with a green channel mechanism, revise resource allocation plans, reduce the time that patients spend in the ICU or operating room, and minimize intermediate steps.

This study revealed that patients transported by ambulance tend to be in more critical conditions than those who are self-admitted. Nursing staff, especially triage nurses, should pay more attention to patients arriving by ambulance, as the appropriate handling of these patients can significantly impact the operational efficiency of the ED. During the pretriage process, it is crucial to quickly identify the severity of a patient’s condition and stratify their risk, allowing for rapid emergency interventions for level I and II patients. Upon entry into the resuscitation room, medical and nursing staff must promptly prepare for resuscitation, implement strict monitoring to quickly identify patient risks, improve the success rate of emergency resuscitation, and reduce mortality rates. Patients transported by ambulance require close monitoring of their conditions during the waiting period, with dynamic adjustments and timely notifications to physicians for possible reclassification, reducing patient risk and ensuring safe nursing care, which positively influences the short-term outcomes of in-hospital patients.

## Acknowledgments

The authors gratefully acknowledge the financial support provided by the Beijing Hospitals Authority Youth Program (code: 2019-YJJ-ZZL-047).

## Author contributions

**Conceptualization:** Yan-Hua Ma.

**Data curation:** Ying An.

**Formal analysis:** Ying An.

**Project administration:** Ya-Mei Guan.

**Resources:** Ya-Mei Guan, Dan-Dan Zhu.

**Software:** Dan-Dan Zhu.

**Validation:** Na Liu.

**Visualization:** Na Liu.

**Writing – original draft:** Xia Ji.

**Writing – review & editing:** Xia Ji.
